# Identifying Settler Colonial Determinants of Health (SCDH) as the Upstream Cause of Palestinian Ill Health Is Both Incorrect and Harmful

**DOI:** 10.5041/RMMJ.10544

**Published:** 2025-04-29

**Authors:** Linda Young Landesman, Maya Korin, Stacey Plichta, Brian Englander, Ora Paltiel, Public Health Professionals Against Antisemitism Working Group

**Affiliations:** 1 Office of Professional Services and Affiliations (Retired), New York City Health and Hospitals Corporation, New York, NY, USA; 2 Department of Environmental Medicine and Climate Science, Icahn School of Medicine at Mount Sinai, New York, NY, USA; 3 Health Policy and Management, City University of New York, New York, NY, USA; 4 Perelman School of Medicine, University of Pennsylvania, Philadelphia, PA, USA; 5 Braun School of Public Health, Hadassah-Hebrew University Faculty of Medicine, Jerusalem, Israel

**Keywords:** Health disparities, health equity, minority and vulnerable populations, public health systems research, settler colonialism, social determinants of health, structural racism

## Abstract

Extremism, displacement, and ongoing conflict have affected Palestinians and Israelis personally and collectively, endangering their health and lives. A theory circulating in public health publications posits that settler colonial determinants of health (SCDH) are the root cause of health inequities in the region. We argue that this assertion is misleading, ignores key facts, and exacerbates polarization, thus harming health. Public health is an evidence-based, scientific discipline based on hypotheses, research, and analysis. Throughout the scientific process, careful assessments of bias are essential. Knowledge is subsequently translated into policy and action. The SCDH theory rejects this approach as tainted by “colonialism.” We also argue that the SCDH concept, as applied to health disparities in Israel-Palestine, is an ideologically driven theory in search of evidence. Rather than developing testable hypotheses, the promoters of SCDH use selective evidence to support its relevance to health in the region. The theory collapses when examined against relevant facts related to regional history and the health status of Israelis and Palestinians. It invokes one-sided racism as a driver of health inequities in a context-inappropriate manner, and ignores many upstream determinants including actions of the Palestinian leadership, and their role as drivers of health. It denigrates peace-building and collaboration which are key to future health and wellbeing in the region, and which have a proven record in improving health outcomes. We call on public health professionals to distance themselves from unfounded rhetoric that polarizes the communities, and undermines the discipline’s scientific integrity, while contributing nothing to promote health in the region.

## INTRODUCTION

### The SCDH Paradigm and Harmful Application to Palestinian Health

Amidst ongoing debates regarding the historical origins of the Israel-Palestinian conflict^1^–^5^ and deliberations whether or not Israel is a “settler-colonial entity,”^5^–^7^ the public health community is increasingly being exposed to claims^8^–^13^ that settler colonial determinants of health (SCDH) are the root cause of poor health outcomes among Palestinians in Gaza, the West Bank, and Palestinian Arab citizens of Israel (PCI). The concept of settler colonialism has been defined as “a system of oppression based on genocide and colonialism, that aims to displace a population of a nation (oftentimes indigenous people) and replace it with a new settler population.”^14^ The SCDH theory leverages the concept that the ultimate, or most upstream, causes of health disparities are structural factors, including the larger socioeconomic context, governmental structures, public policies, and cultural values.^15^ Its proponents allege that “settler colonialism” is even further “upstream” of these factors and thus the ultimate cause of health inequities for indigenous populations.^8^

While a detailed discussion of the origins of the Arab–Israeli conflict and the validity of the settler colonial concept as it applies to Zionism are beyond the scope of this essay, it is necessary to recognize that Jews are viewed by historians as indigenous to the region^16^, as are Palestinians. Jews have a “contractual connection to the land, reiterated in the Bible, collective memory, religious practices and rituals, and cultural appreciation as well as a shared hope and belief in the end of exile and eventual return home.”^7^ Jews have resided in the region continuously since ancient times, and Zionism brought about a resurgence of Jews returning to reside in the land. This essay focuses on the flawed application of the settler colonialism concept to health and particularly to health disparities between Israeli Jews and Palestinian Arabs.

We believe that those who propose that settler colonialism is the root cause of poor health outcomes in the region have misidentified true causes and have failed to identify key indicators.^17^ Having done so prevents improvements in the health of residents of the region through eventual peace and health system strengthening, cooperation, and attention to risk factors which are modifiable. Further, the SCDH paradigm is scientifically flawed because it uses loose and deceptive terms to avoid providing concrete criteria to measure an exposure that could be tested rigorously for its impact on health outcomes.^8^ Yet despite this lack of scientific rigor and evidence, 147 faculty at 77 different schools of public health and medicine publicly committed to teaching their students this theory, and some are currently doing so in the United States (US).^18^–^21^

## A CRITICAL LOOK AT SCDH TERMS AND CLAIMS

An examination of the terms and claims foundational to SCDH theory in the Israel-Palestine context highlights the flawed underpinnings of this theory, as described below:

### Proponents of SCDH Are Ideologically Motivated, Lack Data, and Are Dismissive of the Scientific Basis of Public Health

Settler colonialism is defined in vague terms as an insidious and “shapeshifting” power structure;^22^ its advocates use nebulous, ideologically motivated, and tendentious language to support what they claim are its effects on health for Palestinians and Israelis.

In fact, proponents of the SCDH explicitly oppose the use of empirical evidence and research in public health, thus undermining the validity of their concepts. For example, Wispelwey et al. maintain that there is a need to challenge the foundations of public health, including epidemiological models, research methods, and the building of policy on evidence. They argue that the “gatekeeping role” of the scientific model and those who adhere to it are racist and “infused with coloniality.”^8^

A review of the recent literature of SCDH on PubMed using search terms “settler colonial” AND “health” AND “Palestine” or “Gaza” as applied to health in Israel-Palestine reveals the paucity of science underlying its claims (see Supplement A). Champions of SCDH are dismissive of scientific reasoning and the factual basis for decision-making. They dispute the use of facts as a basis for action and make assertions about causation yet provide no compelling data-based studies that link settler colonialism to health. Furthermore, they argue that such a connection is “undiscoverable” using the scientific method.^8^ They propose that knowledge be “decolonized” from “neoliberal knowledge production by Anglo-Saxon academia, journals, books, international research groups, and funding agencies, among others.”^13^ In short, the proponents of SCDH advocate dispensing with empiricism for the sake of an ideological stance. Instead of data, they offer references in support of SCDH theory which are themselves polemics.^23^ They do this at a time when unsubstantiated conspiracy theories abound about other public health topics, threatening the public’s support for the factual basis of public health policy.

The proponents of this concept generate very little data. When they do, it is generally qualitative. Occasionally they use secondary sources of quantitative data to back their claims, but these are partial. The authors fail to provide alternative explanations for observed health disparities (see Supplement A).

The SCDH proponents clearly have an activist agenda and say so explicitly. They have created an opus in various public health publications which provides volume, but not substance. In what may appear shocking to many public health practitioners and advocates, their ideology is highly critical of the human rights and humanitarian approaches to public health.^24^ They explicitly state that activism should translate into knowledge, rather than the broadly accepted academic paradigm of knowledge acquisition and transfer leading to action.^25^ Many articles include the same authors, who reiterate and rehash similar claims without providing new data. Yet these repetitive claims are put forward as a basis for public health teaching^8^ and have, in fact, been incorporated into public health curricula at Harvard University in the US.^19^–^21^

### SCDH in the Israel-Palestine Context Rejects the Concept that Conflict Is a Cause of Ill Health and Health Inequities

There is no doubt that a century of conflict has had grave effects on the health and wellbeing of Israelis and Palestinians. Further, during times of conflict, governments, like those in Palestine and Israel, expend resources on materiel of war. This diversion of resources limits their ability to spend on basic societal needs including public health. The “peace dividend” would allow more resources to be allocated to social programs and health. Yet, glaringly missing, or disparaged by the SCDH enthusiasts as “colonialist,” are conflict and war as causes of poor health—and peace, regional cooperation, or collaboration as a remedy.

The advocates of SCDH reject the notion that conflict is at the root of many health disparities in the region.^26^ Yet, the tragic consequences of the current war, instigated by the Hamas-planned and executed violent atrocities of October 7, 2023, include the loss of life for thousands of Palestinians, as well as the destruction of health infrastructure, deaths, injuries, and displacement and severe mental health consequences for Israelis and Palestinians. These catastrophes should remind us that war is the most devastating determinant of health.

Settler colonialism, when portrayed as the “cause of the causes” is presented as irremediable and akin to original sin,^6^ except by its antidote—“decolonization”—a threatening solution in the Israel-Palestine context. As such, if settler colonialism is a modifiable determinant of health, then decolonization may entail “freeing Palestine from the river to the sea” or sending the Jews back to Europe and the Middle Eastern countries from which they fled as refugees, or completely eliminating Jews from residing in Israel. These calls are historically concerning rationalizations for the racist violence against Jews which the world has witnessed since October 7, 2023. Of equal concern is the fact that by rejecting conflict as a basis for the health inequalities noted in Israel-Palestine, the settler colonialism “theorists” are implicitly endorsing its prolongation, with disastrous results, such as those we are currently witnessing for Palestinian health and wellbeing.

### SCDH Concepts Use Racialist Theories and Portray the Majority of Israelis as “European” or White

The “settler colonialist” argument as applied to Israel-Palestine frames Israelis as European and as white supremacists. The basic tenet of this argument is that white supremacy and structural racism are at the core of all “settler” governance, institutions, and societal practices.^8^ Wispelwey et al.^8^ state that Israeli Jews claim racial superiority over Palestinian Arabs and have used this to exploit Palestinians. This statement can be viewed as a pernicious form of antisemitic “Holocaust Inversion,”^27^ a tactic used to frame Jews as oppressors. The irony is inescapable given that white supremacists in Nazi Germany and currently in the US both portrayed Jews as a non-white lesser race, providing the rationale for annihilating them during the Holocaust and attacking them to the present day.^28^

In fact, most Israeli citizens are not “white,” nor do they originate from Europe,^29^ and the majority would be considered “people of color” using an American-centric racial lens. A full understanding of the history of Jews in the Middle East needs to encompass the fate of the almost one million Jews who lived in Arab lands (often called Mizrahi Jews) for centuries. After World War II, most Arab states passed laws that led to the complete expulsion, or ethnic cleansing, of their Jewish citizens and the confiscation of their property.^30^ As a result, at least 850,000 of these Jews fled to Israel from countries in North Africa and the Middle East where many had lived for centuries, if not millennia. Those who remained suffered great oppression, and currently fewer than 10,000 remain in Arab lands.^31^ The portrayal of refugees as “colonists” and of Asian, Ethiopian, and North African Jews as “white” again serves ideological purposes but does not reflect historical truth. As such, critical theorists, such as those who advocate for SCDH, are themselves engaging in discrimination when they fail to apply critical theory to the examination of Jewish oppression over the millennia. By framing Zionism as the model for systemic colonialism, rather than a response to oppression, they are “reproducing the system in power dynamics.”^32^

Wispelwey et al. maintain that colonialist settlers, defined by them as “Western, capitalist and modern,” use racialized narratives to craft policies that seek to expropriate Native lands and exploit their labor and resources.^8^ In fact, Jews did not “extract” resources from Israel; the land was of poor quality and had few resources. Prior to the establishment of the modern Israeli state, Zionists invested in the land, applied more advanced agricultural techniques, and emphasized the use of their own labor as a matter of official policy.^33^ Wispelwey et al.^8^ further claim that the Jews who came to Israel, whom they consider “settlers,” came with pre-accumulated wealth to assist them in their efforts; but this does not correspond to the historical reality. Jews came to Israel largely as impoverished refugees forced to flee countries where their property was confiscated and citizenship was denied. These refugees, including those who came from Yemen and Ethiopia after the establishment of the state of Israel,^34^ were joined later by those from the former Soviet Union who were well-educated but did not possess material wealth.

An example promoting the SCDH argument is a recent article in *Health Equity* by Asi et al.,^12^ which asserts that anti-Palestinian racism is the major driver of health inequities. Using jargon with currently fashionable rhetoric but questionable applicability, Asi et al. inappropriately frame their piece in the context of American race relations, Critical Race Theory and Black Lives Matter. They imply that Israelis are “white” when, as noted, the majority are people of color. Although their argument may appeal to proponents of intersectionality, it has little relevance to the Israel-Palestine context. For example, they write: “The structural violence against Palestinians is rooted in settler and indigenous dynamics that are further complicated by anti-Muslim racism serving as mutually reinforcing forms of structural racism.”^12^^(p372)^ However, to make this point, Asi and colleagues cite Samari,^35^ who writes about Islamophobia in the US, which is not applicable to the much more complex levels of health inequity and nationalities of people in the Middle East.

The SCDH theorists frequently use the word “apartheid”^12^,^36^ to describe the health system in Israel. In reality, Israel has a National Health Law guaranteeing equal access and rights to services for all its inhabitants. The Ministry of Health has dedicated departments which measure indicators of quality and programs which monitor health inequalities.^37^ Further, based on data, the providers of primary care in all four Israeli health plans propose and implement programs to minimize inequalities, including remedial actions specifically directed at PCI.^38^ A survey has shown that PCIs have higher levels of trust in the Israeli health care system than other immigrant and non-immigrant Israelis.^39^

Further, the health system is among the most integrated sections of the economy in Israel.^40^ In 2023, Israeli Arabs, who make up 21% of the population, have a significant role in the delivery of health care services in Israel, constituting 25% of physicians, 27% of nurses, and almost 50% of pharmacists.

Despite equal access to health care, racism and discrimination have been observed in Israel, as it has in many health systems, and the issue is addressed both by public health researchers and health care providers. A systematic PubMed literature search with key words [racism AND health care AND Israel] yielded 73 citations, of which 14 were actually relevant dealing with stereotyping, bias, and racially motivated patient preferences in Israel (see Supplement B). This body of work indicates that serious research about and examination of racism related to the Arab minority, migrants, and other ethnic minorities in the health sector in Israel does occur; discussion of these issues does not require the SCDH lens. However, none of these references was cited by Asi and co-workers^11^,^12^ This void raises questions about their level of scholarship. It also elicits concerns that they have intentionally discounted both the introspection and awareness of forms of racism and proposed solutions currently being addressed by the Israeli health sector, and the scientific enquiry that has been carried out by Israeli scholars on this issue.

### The SCDH Argument Fails to Explain Palestinian Demography and Health Outcomes

An examination of both history and current data demonstrates that the application of the SCDH paradigm is incorrect in describing health in Palestine. Even prior to the establishment of the modern state of Israel, strong improvements in the health status and social determinants of health of Palestinian Arabs occurred. This has been attributed, in part, to intensive and successful efforts to eradicate malaria^41^ by Jews who returned to their indigenous homeland in the late 19th and early 20th centuries. As noted by the British Survey of 1950, the standard of living of Arabs in the Middle East greatly increased prior to World War II and the years immediately afterward (1922–1948) coinciding with Jewish immigration to Palestine, with significant drops in infant mortality, marked increases in birth rate, and increases in education level.^42^ Health services in the pre-State Yishuv (Jewish settlements) were available and accessed by Jews and Arabs alike.^43^

Further, before the war in Gaza which followed the October 7, 2023 Hamas attack on Israel, health in the region was also improving for Israelis and Palestinians, Jews, Druze, Christians, and Muslims. This is clear when observing health measures such as life expectancy and infant mortality, health status and social determinants of health, and health outcomes, while the regional demography contradicts accusations of genocide.

#### Life Expectancy and Infant Mortality

One would expect, if the SCDH theory held and was truly the major determinant of health disparities, that all Palestinian citizens of Israel would be affected in a similar way. In fact, among Christian PCI, infant mortality rates are among the lowest in the world (1/1000 live births), even lower than among Israeli Jews, while other PCI, Bedouins living in the Negev, experience nine-fold higher rates.^44^ This suggests that there are clearly other important social determinants of health, besides SCDH. Tautologically, both inferior and superior health outcomes for PCI are falsely attributed to racism by Asi et al., who casually mention that PCIs experience better health outcomes than Palestinians in the occupied territories. Palestinian citizens in Israel now have the longest life expectancy of any Arab Muslim population in the world, and they are outliers compared to their Arab brethren in adjacent countries. The life expectancy of Palestinians receiving health care provided by the Palestinian Authority is remarkably similar to that of neighboring countries, where one can presume anti-Arab racism and settler colonialism do not exist (see Table 1).^45^

**Table 1:** Life Expectancy at Birth for Palestinians under the Palestinian Authority, Palestinian Citizens of Israel, and in Neighboring Arab Countries.

Country^ref.^	Year of Data	Life Expectancy at Birth (Years)	
		Males	Females
Syria^47^	2022	68.7	76.2
Jordan^48^	2022	72.0	76.7
Lebanon^49^	2022	72.4	76.6
Egypt^50^	2022	67.9	72.1
United Arab Emirates^51^	2022	77.7	81.4
Palestinian Authority^52^	2022	71.0	75.9
Palestinian (Arab) citizens of Israel^46^	2022	77.5	82.1
Israel (all)^53^	2022	80.7	84.8

Since the establishment of the State of Israel, life expectancy of both the Arab minority of Israel and that of Israeli Jews has improved, not deteriorated, though gaps have widened.^54^ Furthermore, infant mortality in the Bedouin minority, although still higher than in the Jewish population, has greatly decreased, as has childhood stunting, in part due to special programs initiated by Israel’s Ministry of Health.^55^

#### Health Status and Social Determinants of Health

Health disparities do exist between Palestinian Arabs and Jews, but, when the advocates of SCDH reduce their attribution to a nebulous single cause, they ignore many factors that contribute to ill health in the region. This is clear when behaviors that directly influence health are examined. The PCI and residents of Gaza have high rates of smoking and obesity: >36% of men smoke,^54^.^56^ and obesity is particularly high among women.^54^,^57^ Further, gun violence, with resultant deaths and injury, are higher within the PCI community than in the Jewish community in Israel.^58^ Yet SCDH theory fails to account for how these behaviors contribute to poor health.

#### Health Outcomes

**Figure 1:**
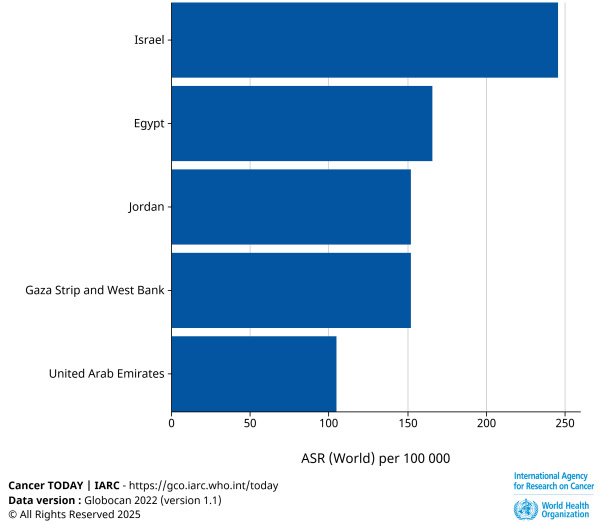
Age-standardized Cancer Incidence in Israel, Palestinian Territories, and Surrounding Middle Eastern Countries. Data are for all cancers. ASR, age-standardized rate. Reprinted from Ferlay et al. (2024),^59^ with permission. Available at: https://gco.iarc.who.int/today (accessed March, 27, 2025).

While increased risks of diabetes, accidents, lung cancer, and cardiovascular disease are seen among PCIs and Palestinians from Gaza and the West Bank compared to Jewish Israelis,^54^ SCDH scholars neglect to mention that both PCIs and these Palestinians have better outcomes in some health indicators than do Jewish Israelis. For instance, cancer incidence and cancer deaths are higher in Israel than in the Palestinian territories, as reflected in the graphic presentation of data from the International Agency for Research on Cancer Age for standardized cancer incidence in Israel, Palestinian territories, and surrounding Middle Eastern countries (see Figure 1)^59^ and higher among Israeli Jews than among Israeli Arabs.^60^ Arab women in Israel undergo screening mammography at a higher rate than their Jewish counterparts, but have less screening for cervical cancer.^61^ Are these disparities also due to SCDH?

#### Regional Demography Contradicts Accusations of Genocide

In August 2023, even prior to the current war in Gaza, Wispelwey and colleagues claimed that Israel was committing genocidal violence.^8^ The demography and health statistics in the region clearly contradict the picture portrayed by the SCDH proponents. In reality, in the entire region of Israel and the Palestinian territories west of the Jordan River, Jews and Arabs each constitute about 50% of the total population. Both the Jewish and Arab population have grown since 1948. Estimates from the US Census Bureau International Database indicate that Gaza’s Palestinian population increased from merely 265,800 in 1960 to over 2.1 million in 2023.^62^

On a global level, according to the Palestinian Central Bureau of Statistics, there were 1.37 million Palestinians in 1948, yet in 2012 the estimated world population of Palestinians totaled 11.6 million.^63^ In contrast, while the Palestinian population has multiplied exponentially, the worldwide Jewish population is smaller than it was before World War II and has not recovered from the Holocaust—an undisputed genocide.^64^,^65^

### SCDH Theory Mimics Public Health Theories Proven False Long Ago

Settler colonial reductionism even minimizes the role of a bacterial infection in the causation and propagation of brucellosis, among the Negev Bedouin. By invoking SCDH theory rather than pinpointing the true cause, which would lead to remediating effects such as ensuring pasteurization of goat milk products, as well as proper food handling, animal husbandry, vaccination, and handling of afterbirths, Tanous and Eghbariah^66^ bring public health back to the dark ages when germ theory was yet to be developed or not yet accepted.

In fact, as a theory “in search of evidence,” the SCDH ideology may be viewed as analogous to the ancient miasma theory of disease and contagion. The latter theory posits that cholera, plague, and other diseases are caused by “bad air” or “stink.” Focusing on stink rather than the true determinants had disastrous health consequences. In 18th-century London, proponents of miasma theory favored diverting London’s waste into its water supply, thus endangering, rather than promoting, the public’s health.^67^ Like the miasma theory, the SCDH framework seeks to explain health outcomes of geographically defined populations subject to an environmental determinant of health. While the miasma theory itself did not explicitly blame specific groups of people for the health issues of others, it did influence public health responses in ways that could harm certain populations. For example, during the Black Death in the 14th century, when the miasma theory reigned, there were efforts to purify the air with smoke and strong-smelling substances. These measures were often intertwined with religious and superstitious beliefs which resulted in Jews being scapegoated and blamed for the spread of the disease in some communities. Analogously, Jews or Israelis are now being blamed for the health status of Palestinians through the SCDH theory, although other explanations, both social and biological, are more plausible.^68^

Both theories may have some basis in common sense—stink is bad—so is colonialism. Miasma’s proponents were frequently well-meaning, and we should presume that SCDH proponents are similarly well-intentioned. Yet the miasma theory, which persisted even after precise causes for specific diseases were known,^69^ was eventually debunked because of its lack of empiricism. Similarly, SCDH fails to meet the rigors of science so crucial to the credibility of public health as a discipline. The erroneous conclusions and application of the SCDH theory in policy decisions has already led to harm. Just like other ideologically based theories that were historically promoted in public health circles, including eugenics, it is false and dangerous and should not be perpetuated. Especially by those committed to advancing the scientific rigors of the public health profession.

### SCDH Arguments Fail to Consider the Main Upstream Determinants of Health in the Region, Notably Political Leadership, Governance, and Weak Health Systems

A serious discussion of health inequities for Palestinians would address governance issues, including corruption in the Palestinian health system.^70^ Unfortunately, Asi and colleagues^12^ appear to exhibit a reverse racism of low expectations and low accountability, absolving Palestinian leaders of responsibility for stunting their own health care system through corruption and misappropriating aid.^71^ They also fail to acknowledge the role of Hamas in harming and jeopardizing the health of Gazans, and the legitimate security concerns of Israel, which have unfortunately resulted in restricted freedom of movement.

The policies and actions of Hamas, a recognized terrorist organization and the elected governing body of Gaza, have resulted in untold harm to the health and daily risks to the lives of Gazan inhabitants. These factors are utterly ignored by the SCDH proponents. Rather than invest in housing and infrastructure, Hamas instigated attacks against Israel which provoked a full-scale war, deliberately used civilians as human shields,^72^,^73^ stole and diverted aid,^74^ destroyed their own people’s access to clean water,^75^ indoctrinated Gazan children to hate,^76^ and built tunnels to shelter fighters but not the civilian population. Claiming that poor Palestinian health outcomes result from “racialization” ignores the fact that Hamas leaders enriched themselves while their population remained impoverished, via smuggling fees, payments from Iran, and possibly theft of aid money.^77^,^78^

Credible scholarship would acknowledge Israel’s legitimate security concerns and government responses, especially when hospitals in Gaza are used as launching sites for rocket attacks,^79^ and ambulances^80^ used to harbor bombs or would-be suicide bombers.^81^ The proponents of SCDH pointedly dispute the role of conflict as a “determinant of health,”^26^ yet the current human catastrophe in Gaza should remind all that war is the ultimate determinant of health, and indeed life.

A serious inquiry into health equity would examine how access-to-care could be addressed or ensured, given (and despite) security concerns. It would also address the role of checkpoints, settlements, and settlement building in restricting access to health services, as well as the threats to health posed by all forms of political extremism. Many of the SCDH writings fail to distinguish health in Israel proper and that in the occupied territories. An exception to the generally vague and evidence-poor writing which advocates SCDH theory is a paper from Fahoum and Abuelaish,^82^ which, while outlining the negative health consequences of settlement-building in hindering health access etc., provides a paragraph explaining that there are other possible explanations for poor health in the region, including issues related to “governance at all levels” and financial burdens on patients and families.^82^ The authors raise legitimate concerns regarding the health of Palestinians under occupation that should prompt the public health community to promote an enduring political solution to the conflict, which would allow two peoples to live in peace and in secure borders. As Abeer Barakat, lecturer at the University College of Applied Sciences in Gaza, said in an interview in the *New York Times*: “We have to accept that Israel exists. And we have to accept the two-state solution. But at least let’s stop the blood bath. Let’s go back to living a normal life.”^83^

Socioeconomic disparities, including the impact on health expenditures, are recognized determinants of health and exist between Israelis and Palestinians, for many reasons. Asi et al.^12^ attribute the 16-fold differences in per capita health expenditures between Israel and the Palestinian Authority to racism, while neglecting to mention that the differences in GDP-per-capita between the jurisdictions are also 16-fold.^84^ Health care financing, and especially access to universal health coverage, are necessary but not sufficient for improving population health and reducing health inequities. Israel has a national health law which guarantees access to care and provision of an essential basket of services to all residents, Jews and Arabs alike.^85^ Rosenthal^86^ described the difference that allocation of governmental resources has made in the public health infrastructure of Palestine and Israel. Yet a closer examination reveals the fragmented nature of health care provision and governance in the Palestinian territories, in part due to severe internal divisions (between Fatah and Hamas). A comparison in Table 2 demonstrates the underfunding and high degrees of reliance on out-of-pocket payments in neighboring countries and in the Palestinian authority. Both variables exacerbate health inequities in these countries despite the absence of settler colonialism (see Table 2).

**Table 2:** Comparison of Health Care Systems and Expenditures in the Middle East Region.

Country	Public Insurance Coverage	Health Care Providers	Governance	Per Capita Health Exp. (US$)	Out-of-Pocket Exp. (%)	% of GDP on Health Care
Israel: Jews and Arabs (PCI)^87^	~100%	Israel MOH Four primary health care plans Private	Israel MOH	3665	19.8	7.9
Palestinian territories – West Bank^88^	76%	Palestine MOH (Palestinian Authority) UNWRA Israeli health plans for East Jerusalem residents Private	Palestine MOH UNWRA Israel MOH (East Jerusalem)	364	33	10.4
Palestinian territories – Gaza^89^	95.4%	Gaza MOH (Hamas) UNWRA NGOs Private	Palestine (Ramallah) MOH Gaza (Hamas) MOH UNWRA	—	—	—
Jordan^90^	64.3%	Jordanian MOH Royal Hospital System UNWRA Private	Jordan MOH	738.5	37.45	7.29
Lebanon^91^,^92^	44% NSSF 18% UNHCR and other	NSSF Private UNHCR	Lebanon MOH NSSF	528.09	34.72	10.06
Egypt^93^	60%	MOH and Population Private	Egypt MOH and Population	615.46	54.9	4.61

Exp., expenditure; GDP, gross domestic product; MOH, Ministry of Health; NSSF, National Social Security Fund; PCI, Palestinian citizens of Israel; UNHCR, United Nations High Commissioner for Refugees; UNWRA, United Nations Work and Relief Agency for the Palestinian People.

### SCDH Theorists Fail to Acknowledge Collaborative Efforts and Programs Which Have Promoted Health Equity in the Region

Historically, efforts where Palestinians and Israelis worked together, such as those involving ministries of health^94^,^95^ and others,^96^–^98^ have improved health outcomes and promoted equity for Palestinians. Between 1967 and the Oslo Accords in 1995, the region was treated as a “single epidemiological unit.”^99^ In the 1970s, vaccinations helped control poliomyelitis in Israel and Palestine through regional cooperation, while tuberculosis, polio, cholera, and measles re-emerged in Syria and neighboring countries during a time of political unrest.^99^ Dismissal of these cooperative efforts as “colonial” is counterproductive and harmful to health.

If historical achievements which were observed when cooperation was supported are an indication, Palestinians and Israelis can create measurable improvements to the health of Palestinians and Israelis by strengthening future public health cooperation. For example, in their work to improve maternal and child health in Palestine, Palestinian scholars Rahim and colleagues make recommendations in which better outcomes can be achieved through cooperation with others.^100^ Their recommendations include: “strengthen community resources for health, such as training health workers,” “implement a human-resource plan that addresses the long-term development of local capacity,” and “expand the midwifery cadre and strengthen their preservice and in-service training.”^100^

In addition, ongoing efforts occurred with formal cooperation between both health authorities and non-governmental organizations. Non-governmental organizations such as Canada International Scientific Exchange Program,^96^ Project Rozana,^98^ and Save a Child’s Heart,^101^ among others, brought Israeli and Palestinian health care providers together to cooperate for better health.

With shared borders, Israel and Palestine share epidemiological risk during outbreaks of infectious disease. Through collaborative programs conducted by Israeli, Jordanian, and Palestinian health and public health professionals, H5N1 avian influenza was contained and an epidemic prevented.^102^ As Dahdal and colleagues^99^ observe, countries, even wealthy ones, cannot protect the health of their populations “without effective and equitable cross-border cooperation.” Unfortunately, official and unofficial policies have hampered scientific and professional collaboration, for fear of “normalizing” the “occupation” of the Palestinian territories. Despite Israelis wanting to work with Palestinian colleagues,^103^,^104^ the Palestinian Authority and Hamas have at times each discouraged or formally forbidden cooperation, and Palestinian scientists have felt threatened if they collaborate with Israeli researchers. Palestinian scientists have also voiced fears for their personal safety if they foster or continue open collaboration with Israeli teams.^105^

During the COVID-19 pandemic, initial cooperation was short-lived as political maneuvering revealed the fragility of these partnerships. Initially, some formal cooperation occurred as public health authorities from Israel and Palestine met regularly for “exchange of medical information and disease outbreak data, lessons on medical management, transfer of vaccines, and the vaccination of cross-border populations.”^99^ When political interference stopped this coordination in July 2020, the ability of Israeli and Palestinian health professionals to work together as a single epidemiological unit was limited.^99^ The lack of continued cooperation was attributed by Qato^106^ to settler colonialism, and by Howard and Schneider^107^ to capitalism and efforts at “elimination,” yet there are alternative explanations, such as unequal capacities of the two health care systems and vaccine nationalism which was observed across many jurisdictions.^108^ Despite early agreements on sharing vaccine, the inability to maintain sustained coordination resulted in a diminished sharing of resources, knowledge, and regional surveillance.^99^

Continued and enhanced cooperation between Israelis and Palestinians, a necessary step to achieve peace, will provide much-needed public health and medical resources which the current public health and health care facilities are not equipped to provide in Gaza and the West Bank. These services include tertiary and quaternary medical interventions and continued treatment in Israeli hospitals of Palestinian patients with advanced illness. The delivery of this much-needed health care requires international efforts and would be facilitated by voluntary activities like Road to Recovery,^109^ and joint efforts of Jewish, Muslim, and Christian clinicians working together to provide clinical care and publish research that improves the health of the region. Advocates of SCDH fail to acknowledge the value and important contribution that such cooperation makes to Palestinian health status.

## DISCUSSION AND CONCLUSION

In this paper, we demonstrate that the SCDH concept does not meet the standards for validity so crucial to scientific integrity. A basic tenet of scientific pursuit in search of truth is the proposal and testing of hypotheses which can be validated or refuted upon examination and re-examination with validated data and tools. The SCDH theory contends that this very approach is itself an expression of settler colonialist oppression and therefore should be rejected in favor of ideologically driven assertions which need not or even should not be subject to such scrutiny. As a specific and especially revealing example of the inherent dangers of this formulation, we describe how the “settler colonial” framework, as it relates to the health of Israelis and Palestinians, is not only unhelpful, it is antithetical to public health as a scientific discipline. The concept is disingenuous, historically inaccurate, and untestable in the context of Israel-Palestine. It fails to provide explanations which would help us understand why Palestinians have poorer health. Rather, the theory is primarily being used to delegitimize Israel, even though Israel is a leader in many arenas of health care and public health.

Theoretical frameworks are important and may help to both conceptualize and provide perspectives to public health research. As Gauffin and Dunlavy point out, “explicit utilisation of theory is crucial to further the development of public health as an academic discipline,” yet the theories themselves must be ethical and grounded, and not, like eugenics and miasma, harmful to health. It is our duty as public health and health care professionals to reject biased, ideologically driven, and baseless theories like the SCDH concept which demonize one group–Jews and Israelis–as the sole determinant of another group’s health.^110^

The SCDH theory promotes ideologically driven and ethically objectionable ideas as scientific givens. Moreover, as a political ideology, SCDH presents specific dangers to Palestinian health. Embracing the fallacious theory of SCDH, specifically the program of decolonization, discourages both serious scholarship and cooperation on initiatives that can improve public health for both Palestinians and Israelis.^111^ It is a divisive ideology at a time when finding common ground is crucial in order to move forward to a resolution of the intractable conflict between Israelis and Palestinians, a requisite for health.

Endorsing SCDH ignores the other critical determinants of health in the region. Rather than distracting the public health community with unscientific, unvalidated, and unprovable concepts, efforts should be made to encourage mutual recognition of the right to health and safety for Palestinians and Israelis alike. Critically, the SCDH theory misattributes many of the causes of health inequities experienced by Palestinians rather than attribute any responsibility to the Palestinian leadership. By failing to address disparities in income, education, health behaviors, and health literacy (which have little if anything to do with settler colonialism), the SCDH concept fails to account for key contributors of health inequalities. By polarizing scientific discourse, it also impedes constructive discussion on how to minimize discrimination and promote equitable access to health care. Further, by framing Israel as a white supremacist and racist project^8^ alongside other clear examples of colonialism, the SCDH theory threatens the 7 million Jews living in Israel today. The delegitimization of Israel, the only Jewish nation among 195 recognized countries, frighteningly echoes the erasure espoused in the Hamas charter which threatens all Jewish lives.^112^ As public health practitioners we are obligated to discover, name, and seek to address the modifiable determinants of health inequalities in Israel and Palestine (including governance and conflict) in order to improve the health of all inhabitants of the region.

As a theory, SCDH is also harmful to public health in the region. It polarizes the scientific community^113^ and jeopardizes the chance for both improved health and peace-building by discouraging dialogue, “normalization,” clinical and training cooperation, and much-needed research ties between Israeli and Palestinian health scientists. Rather than encouraging efforts towards collaborative pursuits, the SCDH advocates denigrate and disparage these activities. Joint efforts to combat infectious disease, prevent and treat chronic disease, and mitigate the health effects of climate change, all of which are highly relevant in the region, would be useful contributions to public health and, arguably, peace.^111^

As public health professionals seeking health equity, we condemn all forms of racism, including Islamophobia and anti-Semitism. In seeking justice, we must eschew polarization, hate, erasure, and a perpetuation of violence. Achievement of health equity is better served by an honest rendering of the sources of health disparities, concerted efforts to rebuff extremism and rejectionism, and advocating steps toward coexistence and a shared future for Palestinians and Israelis. We call on the public health community to move towards peace, cooperation, and recognition of the mutual pain caused by this ongoing conflict, and do so by offering practical and humane solutions to promote health for all in the region.

## Supplementary Information



## Abbreviations

GDP: gross domestic product
PCI: Palestinian citizens in Israel
SCDH: Settler Colonial Determinants of Health
US: United States
