# Supplementary material for: Identifying Settler Colonial Determinants of Health (SCDH) as the Upstream Cause of Palestinian Ill Health Is Both Incorrect and Harmful
Source: Rambam Maimonides Med J. 2025 Apr 29;16(2):e0009. doi: 10.5041/RMMJ.10544 (PMC12053806; doi:10.5041/RMMJ.10544)
Supplement: Supplementary file 1 [file rmmj-16-2-e0009_Supplement.pdf]

SUPPLEMENTARY MATERIAL

This appendix has been provided by the authors for the benefit of readers

Supplement to Identifying Settler Colonial Determinants of Health (SCDH) as the Upstream Cause of Palestinian Ill Health Is Both Incorrect and Harmful

Landesman LY, Korin M, Plichta S, Englander B, Paltiel O, Public Health Professionals Against Antisemitism Working Group. Identifying Settler Colonial Determinants of Health (SCDH) as the Upstream Cause of Palestinian Ill Health Is Both Incorrect and Harmful. Rambam Maimonides Med J 2025;16 (2):e0009. Review. doi:10.5041/RMMJ.10544

SUPPLEMENT A

This supplement provides a table summarizing our literature review (following pages).

Supplement A: Table 1. Literature Review and Comments on Articles on Settler Colonial Determinants of Health of Israelis and Palestinians. Source: PubMed search: "settler colonial" AND "health" AND "Palestine" OR "Gaza."

First Author (Year)REF	Context for Settler Colonialism	Study Type	Health Condition Addressed	Main Argument and Illustrative Quote	Alternative Explanations for Health Inequities	Comments
Sousa et al. (2019)[1]	Place as a SCDH, restricted access as a manifestation of SC	Qualitative based on focus groups	"Well-being" and access to health care and facilities, among others	"Analytically, [the article] is informed by critical place inquiry, which … focuses centrally on the geographic and spatial dynamics of colonisation, and particularly SC, as key determinants of individual and collective well-being."	None offered	Conflict and security concerns in addition to settlement expansion
Fahoum and Abuelaish (2019)[2]	How settlement construction in West Bank impacts health (Note SC is only in key words, raising a question on whether the authors used it or editors added it)	Historical review, with data	Access to health care, exposure to political violence, economic conditions, and water contamination	"Settlement construction and associated policies have several downstream consequences that impact various aspects of life in the West Bank, including mobility restrictions; employment; economic well-being; access to health care, clean water, housing and agricultural land; and exposure to political violence."	Study points out that it does not address individual determinants of health. Also—governance, trade and relationships with surrounding countries, financial burden on low-income patients	Only study pointing out other plausible explanations for poor health status. These do not have to be mutually exclusive. Clearly, settlement activity and checkpoints diminish access to health and other services
Qato (2020)[3]	Settler colonialism precedes and is fundamental to all other determinants of health	Essay	Health inequalities, Covid-19	Essay tackles "the implications for health and health research of eliding ongoing settler colonialism"—everything falls under that rubric	Palestinian Authority cooperation with Israel on Covid-19 response deemed "SC tools"	Article critiques the epidemiologic approach, data acquisition and theories or arguments not subsumed by SCDH. Reductionist approach. SC explains all
Bouquet et al. (2022)[4]	Structural racism and apartheid based on SC	Historical review	Health in general; life expectancy and infant mortality gaps mentioned	"The radical dismantling of systematic racial oppression and domination in Palestine, tantamount to apartheid, is a precondition for realizing the right to health for all."	None offered	Health behaviors, smoking, health system gaps, risk factor control not mentioned; reductionist

First Author (Year)[REF]	Context for Settler Colonialism	Study Type	Health Condition Addressed	Main Argument and Illustrative Quote	Alternative Explanations for Health Inequities	Comments
Majadli & Ziv (2022)[5]	Israel's narrative of "securitization" shifts the focus from the root cause: SC	Two case studies and discussion	Limb amputations of protesters on the Gaza border	[Palestinian] "right [to health] was never guaranteed but always conditioned on Israel's definition of its security needs"	Security concerns	Consequences of ongoing conflict and suspicion resulting in unfairness, curtailing the right to health
Asi et al. (2022)[6]	Historical necessity for public health practitioners to target the SC that lies "beyond [the] 'causes of causes'" of Palestinian ill health	Editorial	General	Perspectives, examples, and description of settler colonialism's "strategies of elimination" are greatly needed	Security issues, humanitarian approaches and conflict are dismissed in favor of a SC approach	This article frames SC as *the* cause of Palestinian ill health to the exclusion of other explanations. The paper does not include supporting data
Tanous & Eghbariah (2022)[7]	How colonial and SC policies have targeted Palestinians and their goats and contributed to the distribution of brucellosis along ethno-national lines	Historical review	Brucellosis, much more common among Palestinian citizens of Israel, especially in the South	"it is crucial to move beyond blaming marginalized and colonized communities for their behaviors or cultures, in itself an act of symbolic violence, and address the historical injustices toward humans and goats"	Cultural practices, unpasteurized milk	Reductionist with no attribution to personal responsibility
Howard & Schneider (2022)[8]	Israel's public relations efforts as a lens to examine the types of ideological frameworks that sustain SC amid rhetorical commitments to equality and health	"Critical discourse analysis"	Lack of access to Sars-CoV2 vaccines	"settler-colonial societies use liberal frameworks of citizenship and capitalism to carry out their racialized projects of elimination"	None considered	Wider context: vaccine nationalism as a widespread phenomenon, scarce resources
Joronen (2023)[9]	Examples drawn from the colonial context of Palestine … terrorizing the everyday life	Personal narrative and philosophic discussion	"Weaponization of breathing" due to use of tear gas, skunk water	Palestinians adapt their breathing to violent circumstances; "they learn to breathe in rhythms different to those of occupation, so making breathing a transformative moment—a moment of transforming the atmospheres of colonial violence."	Not addressed	Security concerns—use of tear gas and skunk water preferable to more lethal weapons

First Author (Year)[REF]	Context for Settler Colonialism	Study Type	Health Condition Addressed	Main Argument and Illustrative Quote	Alternative Explanations for Health Inequities	Comments
Veronese et al. (2023)[10]	Transgenerational psychosocial impacts of colonization [the Nakba] and the structural and cultural violence against indigenous populations	Qualitative, semi-structured key informant interviews	Mental health, resilience, quality of life	"Memories of the forced migration mirrored in the three interviewed generations through idioms such as faji'ah (the calamity) and jalwa (to transfer) are shared in the Palestinian narrative of collective trauma and reinforced by the ongoing experience of SC violence."	Mental health humanitarianism in Palestine does not explain the profound political roots of the collective suffering	Multigeneration refugee status perpetuating intergenerational trauma
Wispelwey et al. (2023)[11]	1. SC is enduring;<br>2. SC "adds explanatory power to racism's health impacts" …<br>3."… advances in structural racism methodologies to elucidate the possibilities for a body of literature linking SC and health, illuminating future research opportunities and pathways toward the decolonization required for health equity"	Perspective	Health inequities in general	SC is a/the principal upstream determinant of health.<br>"we offer the settler colonial determinants of health as an analytic framework ripe for questioning, studying, and challenging health inequities"	None offered	A position paper making a fervent call to use the SC paradigm for research and teaching but providing no compelling argument why.<br>We address this article most thoroughly in the text of the paper.
Tanous et al. (2023)[12]	"When utilising a critical analysis of the root causes of health inequity in Israel—SC and resultant structural racism—the inadequacy of many existing explanations for worse health outcomes becomes clear"	History<br>Secondary data	Health inequalities for Palestinian Citizens of Israel (Israeli Arabs)	Structural racism as *the* root cause. "Acknowledging and redressing the racist policies and practices impacting PCI is a prerequisite for achieving complete health equity in Israel"	None offered	Disparages minority status, ethnicity, and religion as determinants, in favor of structural racism

First Author (Year)[REF]	Context for Settler Colonialism	Study Type	Health Condition Addressed	Main Argument and Illustrative Quote	Alternative Explanations for Health Inequities	Comments
Giacaman (2023)[13]	Calls for the decolonization of knowledge production	Opinion and historical review	Knowledge about health	Palestinians have been "colonized by the imposed knowledge, theories, interpretations, and methodologies" of Anglo and neoliberal communities	—	Public health as a scientific discipline is at risk if knowledge and data are denigrated
Engebretsen & Baker (2024)[14]	Calls for decolonizing global health have ultimately proved no more than window dressing	Editorial	Destruction of health facilities; Global health as a discipline	The "decolonization approach has failed to address the enduring violence and oppression perpetuated by the global political economy, which is underpinned by colonial and capitalist ideologies." Advocates for confronting [decolonization] with an action-to-knowledge strategy rather than a knowledge-to-action strategy	"choosing … to treat the aftermath of colonial violence as a humanitarian crisis" (as done by the WHO)	Disparaging knowledge in favor of rhetoric and ideology
Veronese et al. (2024)[15]	Global health, as currently practiced, is not merely ineffective but complicit in maintaining settler colonial structures, particularly in Gaza, where health inequities are deeply intertwined with the ongoing occupation	Editorial and commentary on Engebretsen et al.[14]	"Health inequities" and health crisis in Gaza	"The root cause of the health crisis is the fact that Palestinians were dispossessed of their land in 1948." [Accusing Hamas of barbarism] reinforces "harmful colonial stereotypes of Palestinians." Framing the issue as a conflict "reduces Gaza's health crisis to an issue of internal fault rather than recognizing the external forces—namely SC and occupation—that systematically dismantle its health infrastructures."	—	Hamas violence is not mentioned or discussed as a "cause" of the health crisis in Gaza. It is all due to dispossession in 1948

First Author (Year)[REF]	Context for Settler Colonialism	Study Type	Health Condition Addressed	Main Argument and Illustrative Quote	Alternative Explanations for Health Inequities	Comments
Asi et al. (2024)[16]	Palestinian health inequities are rooted in settler colonialism and racism	Perspective	Health inequities: life expectancy, infant and maternal mortality rates, and greater risk of chronic disease	"Recognizing structural racism as a health equity threat to Palestinians should be a natural extension of an emerging consciousness about what is needed to dismantle structures of oppression"	—	See commentary in text
Sharma (2024)[17]	Targeting health care systems is a method of settler colonialism	Commentary	Health systems<br>Destruction in Gaza War	"While the global health narrative continues to divorce Palestinian health from intersections of violence and historical trauma born out of Israeli settler colonialism, we need to contextualize how targeting healthcare systems is inherently a practice of systemic oppression."	—	This is an example of the export of the SC argument—author is Indian and so is the journal of publication. In other words, SCDH theory is gaining traction globally
Tanous & Hagopian (2025)[18]	Housing is a fundamental determinant of health. SC in the US and Palestine impinge on this right	Review	Housing as a SCD	"Homelessness is not a reflection of personal choices but rather the malicious byproduct of structural racism and oppression."	Housing as a human right	No acknowledgement of conflict. Conflation of US and Palestinian root causes of homelessness
Smith et al. (2025)[19]	Must address the SC root causes and not take a neutral "humanitarian" approach	Editorial	Rebuilding the health system in Gaza. A critique of Blanchet et al. (in Confl Heal 2024;18:42)	Plan for rebuilding Gaza health system inadequately addresses root causes including SC	—	Critique of humanitar-ianism. SC demanding more activist approach and sanctioning "resistance"

SC, settler colonialism; SCD, settler colonial determinant; SCDH, settler colonial determinants of health.

References to Supplement A

1. Sousa CA, Kemp SP, El-Zuhairi M. Place as a social determinant of health: narratives of trauma and homeland among Palestinian women. Br J Soc Work 2019;49:963–82. CrossRef
2. Fahoum K, Abuelaish I. Occupation, settlement, and the social determinants of health for West Bank Palestinians. Med Conf Surviv 2019;35:265–83. CrossRef
3. Qato DM. Introduction: Public health and the promise of Palestine. Journal of Palestine Studies 2020;49:8–26. CrossRef
4. Bouquet B, Muhareb R, Smith R. "It's not whatever, because this is where the problem starts": racialized strategies of elimination as determinants of health in Palestine. Health Hum Rights 2022;24:237–54. PMID: 36579312
5. Majadli G, Ziv H. Amputating the body, fragmenting the nation: Palestinian amputees in Gaza. Health Hum Rights 2022;24:281–92. PMCID: PMC9790951
6. Asi YM, Hammoudeh W, Mills D, Tanous O, Wispelwey B. Reassembling the pieces: settler colonialism and the reconception of Palestinian health. Health Hum Rights 2022;24:229–35. PMID: PMC9790940
7. Tanous O, Eghbariah R. Organized violence and organized abandonment beyond the human: the case of brucellosis among Palestinians in Israel. mSystems 2022;7:e0149921. CrossRef
8. Howard N, Schneider E. COVID-19 vaccination in Palestine/Israel: citizenship, capitalism, and the logic of elimination. Health Hum Rights 2022;24:265–79. PMCID: PMC9790952
9. Joronen M. Atmospheric negations: weaponising breathing, attuning irreducible bodies. Environ Plan D 2023;41:765–83. CrossRef
10. Veronese G, Mahamid F, Bdier D. Transgenerational trauma and collective resilience: a qualitative analysis of the experiences of settler-colonial violence among three generations of Palestinian refugees. Int J Soc Psychiatry 2023;69:1814–24. CrossRef
11. Wispelwey B, Tanous O, Asi Y, Hammoudeh W, Mills D. Because its power remains naturalized: introducing the settler colonial determinants of health. Front Public Health 2023;11:1137428. CrossRef
12. Tanous O, Asi Y, Hammoudeh W, Mills D, Wispelwey B. Structural racism and the health of Palestinian citizens of Israel. Global Public Health 2023;18:2214608. CrossRef
13. Giacaman R. Palestinians under Israeli settler colonialism and "Anglo-centric" colonization of knowledge production. Int J Soc Determinants Health Health Serv 2023;53:146–53. CrossRef
14. Engebretsen E, Baker M. The rhetoric of decolonizing global health fails to address the reality of settler colonialism: Gaza as a case in point. Int J Health Policy Manag 2024;13:8419. CrossRef
15. Veronese G, Kagee A, Jamei YA. Confronting the colonial roots of global health inequities in Gaza: comment on "The rhetoric of decolonizing global health fails to address the reality of settler colonialism: Gaza as a case in point." Int J Health Policy Manag 2024;13:8768. CrossRef
16. Asi YM, Sharif MZ, Wispelwey B, Abuelezam NN, Ahmed AK, Samari G. Racism as a threat to Palestinian health equity. Health Equity 2024;8:371–5. CrossRef
17. Sharma P. Under siege: the role of settler colonialism in targeting healthcare systems. Indian J Med Ethics 2024;9:207–9. CrossRef
18. Tanous O, Hagopian A. The locations of Palestine and the U.S. in the global map of homelessness: Part 2. Int J Soc Determinants Health Health Serv 2025;55:5–15. CrossRef
19. Smith J, El-Solh S, Hanbali L, et al. Realising health justice in Palestine: beyond humanitarian voices. Confl Health 2025;19:7. CrossRef

SUPPLEMENT B

List of papers resulting from a Pubmed search using the terms “racism” AND “health care” AND “Israel”

1. Halamish-Leshem R, Bokek-Cohen Y, Tarabeih M, Azuri P. Patient racism toward nurses in a divided society: the case of Jews and Arabs in Israel. J Nurs Scholarsh 2024;56:843–53. doi: 10.1111/jnu.13006. Epub 2024 Jul 15. PMID: 39010188.

2. Shai A. Racism and tolerance in live kidney donation in Israel-response to Nesher et al. Am J Transplant 2024;24:1512–13. doi: 10.1016/j.ajt.2024.02.031. Epub 2024 Mar 8. PMID: 38461882.

3. Bokek-Cohen Y. Does racism toward nurses increase as treatment invasiveness rises? Nurs Inq 2023;30:e12547. doi: 10.1111/nin.12547. Epub 2023 Feb 8. PMID: 36755498.

4. Korn L, Bonny-Noach H, Tesler R, Koren G, Nissanholtz-Gannot R. Risk and protective factors associated with multiproblem behaviours of Ethiopian young adults in Israel. Health Soc Care Community 2022;30:e1157–69. doi: 10.1111/hsc.13523. Epub 2021 Jul 27. PMID: 34312930.

5. Daoud N, Abu-Hamad S, Berger-Polsky A, Davidovitch N, Orshalimy S. Mechanisms for racial separation and inequitable maternal care in hospital maternity wards. Soc Sci Med 2022;292:114551. doi: 10.1016/j.socscimed.2021.114551. Epub 2021 Nov 8. PMID: 34763969.

6. Weiss CM. Diversity in health care institutions reduces Israeli patients’ prejudice toward Arabs. Proc Natl Acad Sci U S A 2021;118:e2022634118. doi: 10.1073/pnas.2022634118. PMID: 33790013; PMCID: PMC8040664.

7. Attal JH, Lurie I, Neumark Y. A rapid assessment of migrant careworkers’ psychosocial status during Israel’s COVID-19 lockdown. Isr J Health Policy Res 2020;9:61. doi: 10.1186/s13584-020-00422-0. PMID: 33138855; PMCID: PMC7605873.

8. Ayalon L. Perceived discrimination and stigma in the context of the long-term care insurance law from the perspectives of Arabs and the Jews in the North of Israel. Int J Environ Res Public Health 2019;16:3511. doi: 10.3390/ijerph16193511. PMID: 31547132; PMCID: PMC6801376.

9. Keshet Y. Ethnic discordance: why do some patients prefer to be treated by physicians from other ethnic groups? Soc Sci Med 2019;235:112358. doi: 10.1016/j.socscimed.2019.112358. Epub 2019 Jun 7. PMID: 31196576.

10. Daeem R, Mansbach-Kleinfeld I, Farbstein I, et al. Barriers to help-seeking in Israeli Arab minority adolescents with mental health problems: results from the Galilee study. Isr J Health Policy Res 2019;8:45. doi: 10.1186/s13584-019-0315-7. PMID: 31122285; PMCID: PMC6532130.

11. Keshet Y, Popper-Giveon A. Patient demands for ethnic-based separation in public hospitals in Israel: patients’ and practitioners’ perspectives. Isr J Health Policy Res 2018;7:44. doi: 10.1186/s13584-018-0237-9. PMID: 30428927; PMCID: PMC6236991.

12. Daoud N, Gao M, Osman A, Muntaner C. Interpersonal and institutional ethnic discrimination, and mental health in a random sample of Palestinian minority men smokers in Israel. Soc Psychiatry Psychiatr Epidemiol 2018;53:1111–22. doi: 10.1007/s00127-018-1531-0. Epub 2018 May 17. PMID: 29774378.

13. Keshet Y, Popper-Giveon A. Race-based experiences of ethnic minority health professionals: Arab physicians and nurses in Israeli public healthcare organizations. Ethn Health 2018;23:442–59. doi: 10.1080/13557858.2017.1280131. Epub 2017 Jan 19. PMID: 28100067.

14. Shwartz N, Shoahm-Vardi I, Daoud N. Postpartum depression among Arab and Jewish women in Israel: ethnic inequalities and risk factors. Midwifery 2019;70:54–63. doi: 10.1016/j.midw.2018.12.011. Epub 2018 Dec 14. PMID: 30579099.
